# Effects of Polyphenols on Insulin Resistance

**DOI:** 10.3390/nu12103135

**Published:** 2020-10-14

**Authors:** Gary Williamson, Katherine Sheedy

**Affiliations:** Department of Nutrition, Dietetics and Food, School of Clinical Sciences at Monash Health, Faculty of Medicine, Nursing and Health Sciences, Monash University, BASE Facility, 264 Ferntree Gully Road, Notting Hill, VIC 3168, Australia; katherine-s@live.com.au

**Keywords:** polyphenol, starch digestion, glucose, postprandial, GLUT4, akt, diabetes

## Abstract

Insulin resistance (IR) is apparent when tissues responsible for clearing glucose from the blood, such as adipose and muscle, do not respond properly to appropriate signals. IR is estimated based on fasting blood glucose and insulin, but some measures also incorporate an oral glucose challenge. Certain (poly)phenols, as supplements or in foods, can improve insulin resistance by several mechanisms including lowering postprandial glucose, modulating glucose transport, affecting insulin signalling pathways, and by protecting against damage to insulin-secreting pancreatic β-cells. As shown by intervention studies on volunteers, the most promising candidates for improving insulin resistance are (−)-epicatechin, (−)-epicatechin-containing foods and anthocyanins. It is possible that quercetin and phenolic acids may also be active, but data from intervention studies are mixed. Longer term and especially dose-response studies on mildly insulin resistant participants are required to establish the extent to which (poly)phenols and (poly)phenol-rich foods may improve insulin resistance in compromised groups.

## 1. Insulin Resistance

### 1.1. Characteristics of Insulin Resistance

Insulin resistance (IR) is a deceptively complex condition characterised by impaired insulin responsiveness in target tissues, causing the β-cells in the pancreas to continue to produce extra insulin, leading to eventual malfunction through oxidative stress. The lack of response of tissues to insulin results in a state of transient and unpredictable hyperglycaemia and hyperinsulinemia, together with an inflammatory signature that predisposes an individual to metabolic syndrome and type 2 diabetes (t2D) [1,2]. IR is a hallmark of obesity and of a sedentary lifestyle, driven by excess energy consumption, lack of exercise and certain genetic factors [3]. There is a complex interplay between several physiological processes that conspire together to facilitate development of IR, including an unbalance in lipid metabolism [4], dysbiosis [5,6], chronic inflammation, and misfiring of intracellular signalling pathways [7]. However, deciphering what is ‘cause’ of IR and what is ‘effect’ has proven much more difficult and conflicting opinions exist [4,8]. IR often precedes t2D, and the two conditions are inter-related. The course of t2D development is mainly characterized by declining β-cell function and worsening of IR [9].

In healthy individuals, the release of insulin from pancreatic β-cells drives cellular glucose uptake from the bloodstream into tissues via relocation of the glucose transporter solute carrier family 2, facilitated glucose transporter member 4, SLC2A4 (GLUT4) to the cell surface. This action removes elevated postprandial glucose from the blood, a normal process after eating carbohydrate. However, excess insulin production caused by a habitual high glycaemic index diet leads to elevated binding to the insulin receptor, driving fatty acid synthesis in addition to the normal glucose uptake. All of these actions are dependent on a cascade of phosphorylation events, resulting in the activation of two pathways in particular: the phosphoinositide 3-kinase (PI3K) pathway and the mitogen activated protein (MAP) kinase pathway [10]. Dysregulation of the protein kinase B (akt) pathway most dramatically affects IR and can occur in adipose, liver and skeletal muscle, the three main tissue types that contribute to the phenomenon of IR. Obesity often leads to decreased akt activity by complex multifactorial mechanisms, which are incompletely understood [8]. However, decreased akt activity results in reduced GLUT4-mediated glucose uptake within adipose tissue and skeletal muscle [11].

### 1.2. Measurement of Insulin Resistance

Estimation of IR has been the subject of many publications and there are many models and opinions on the subject, which are beyond the scope of this review. The gold standard to which other methods are compared is the glucose or insulin clamp techniques [12], but these are time consuming, expensive, technically demanding and not practical for most hospitals, labs or other organisations. There are several less difficult ways to estimate IR, using various permutations of blood glucose and insulin concentrations, either fasting or after an oral glucose tolerance test (OGTT). One of the most commonly used is the homeostasis model assessment (HOMA) of insulin resistance (HOMA-IR), requiring only fasting blood glucose and insulin measurement. It is often applied to large epidemiological studies as only these two values are required [13], and a subsequent modification is HOMA-β (also called HOMA2) using a modified equation but the same input parameters [14]. An alternative is the quantitative insulin sensitivity check index, abbreviated to QUICKI, illustrating the quality of scientific humour; this also only requires fasting plasma glucose and insulin concentrations [15]. The Matsuda index is one of the estimations that is derived from the fasting glucose and insulin concentrations, together with their values after an OGTT [16]. The diagnosis of IR can be affected by the method chosen, mostly based on variations in cut-off values [17]. These discrepancies demonstrate that IR is a gradual process and a continuum, with the cut-off value defined in a somewhat arbitrary manner. Triglycerides can also act as a surrogate marker of IR [18]. Anything that modulates one of these indices is considered to have the potential to modify IR, but a measured improvement will not necessarily change the diagnosis from “IR” to “non-IR”, based on the truly continuous nature of the values. Measurement methods of IR have been reviewed and the choice of method is often determined by practicality, cost and ease of measurement in the clinic [19]. Insulin sensitivity is effectively the opposite of resistance; in simple terms, if low amounts of insulin are required to lower blood glucose, then insulin sensitivity is high and insulin resistance is low. The importance of IR as a risk factor for chronic diseases is well documented. For example, epidemiological studies have shown an association between IR as measured by HOMA-IR and risk of cardiovascular disease [20,21].

### 1.3. The Role of Chronic Inflammation in Insulin Resistance

Chronic inflammation is a well-characterised link between obesity and development of IR [2,3]. With obesity, nuclear factor kappa-light-chain-enhancer of activated B cells (NF-kB)-driven pro-inflammatory cytokine secretion by adipocytes and immune cells is enhanced [22]. This leads to the recruitment and accumulation of adipose tissue macrophages, which produce more pro-inflammatory cytokines, including tumour necrosis factor (TNF)α and interleukin-6 (IL-6), that act as feed-forward mechanisms to exacerbate chronic inflammation, dysfunction of adipose tissue, decreased insulin sensitivity and IR [23,24,25,26,27]. The activation of inflammatory pathways has additionally been described in the liver [28,29] and muscle [10,29,30]. TNFα-signalling can also inhibit insulin receptor substrate-1 (IRS-1), attenuating its ability to propagate downstream insulin signalling [24]. Continuous over-consumption of energy can also lead to increased reactive oxygen species (ROS) production with endoplasmic reticulum stress, affecting the ability of β-cells to secrete insulin, and further attenuating inflammation and oxidative stress [31].

## 2. Factors that Affect IR

### 2.1. Macronutrient Intake

Long-term consumption of carbohydrate can affect IR, with significant reductions following intake of whole grain foods [32,33,34,35,36] and increases in IR with a habitual high glycaemic index diet [37]. The latter mediated changes in adiposity and central fat redistribution commonly observed in subjects with IR [38]. Concentrations of polyunsaturated fatty acids, such as n-3, have been found to be beneficial to insulin sensitivity in obese and overweight populations in some studies [39], but a Cochrane review found no overall significant effect of n-3 fatty acids on glycaemic control or fasting insulin [40]. Short term high fat diets (3 days) can induce transient IR [41,42], although results from longer time high fat diets, from 3 days to 4 weeks, were mixed and found either no change in insulin sensitivity [43,44] or a decrease [45]. Differences were also observed between ethnicities, with high fat, high energy diets having a greater effect in inducing IR in South Asians compared to Caucasians [46].

### 2.2. Lipid Metabolism

Lipid metabolism is disturbed in the IR state, partly due to the presence of increased circulating fatty acids and/or accumulation of lipids in the muscle and the liver [4] with activation of inflammatory pathways, inhibition of insulin receptor signalling and changes in the expression of genes that influence insulin action [47]. Hepatic IR is caused by the accumulation of lipids within the liver, rather than visceral fat accumulation [48,49]. Following a high fat diet or overfeeding, the accumulation of lipid outside of adipose depots can increase hepatic gluconeogenesis and disrupt GLUT4-mediated glucose transport in response to insulin in the muscle [4], leading to hyperglycaemia, stimulating β-cells to release further insulin and increase lipogenesis [8].

### 2.3. Gut Microbiota and Exercise

Obesity, a high-fat diet or both can lead to dysbiosis, an imbalance in the gut microbiota. This condition leads to increased intestinal permeability, with pro-inflammatory metabolites, such as liposaccharide (LPS) and other bioactive metabolites reaching the blood and ultimately acting on adipose and other insulin-responsive target tissues to promote IR [6]. LPS can act on the liver to promote triglyceride accumulation and secretion of pro-inflammatory cytokines leading to IR [6,50]. Structured mixed exercise can reduce IR [51], even in younger people [52], and reduce the risk of developing t2D.

## 3. Dietary (Poly)phenols

### 3.1. Sources and Intake

The nomenclature of (poly)phenols is complex and guidelines have been reported [53,54]. (Poly)phenols are made by plants for a variety of functions, including protection against stress, UV absorption and resistance to attack by pests. As a consequence, various classes of (poly)phenols are found in the diet and consumed on a regular basis. The different classes, structures and food sources have been extensively reported elsewhere and the reader is refereed to several comprehensive reviews [55,56] and to the phenol-explorer database [57].

### 3.2. Metabolism of (Poly)phenols

The absorption, metabolism, excretion and bioavailability of (poly)phenols has been reported in many reviews e.g., [55,58]. (Epi)catechins are absorbed in the small intestine, and reach the circulation mostly in a conjugated form [59]. Quercetin occurs as a conjugated form in plants and foods, and the attached sugar is removed before absorption, by lactase phloridzin hydrolase in the small intestine or by the gut microbiota [60]. Anthocyanins and procyanidins are poorly absorbed intact, but their catabolic breakdown products are well-absorbed [58]. Phenolic acids are absorbed, but bioavailability is improved by the action of the gut microbiota [61]. In general, (poly)phenols must reach their target site in order to be active. To affect carbohydrate digestion and intestinal glucose absorption, which occur in the gut lumen, (poly)phenols do not need to be absorbed, but to affect other sites, such as muscle, adipose and cells, the (poly)phenol or its active catabolites must reach the circulation in order to exert activity on the tissue.

### 3.3. General Epidemiology on (Poly)Phenol-Rich Foods and IR/t2D Risk

Epidemiological and human intervention studies are considered here when (poly)phenols, or (poly)phenol-rich foods, have an effect on fasting glucose or fasting insulin, and hence affect IR. Consumption of a ‘healthy diet’ pattern, high in vegetables, fruit and wholegrains, and, therefore, (poly)phenols, can lower t2D risk by 14% [62]. A European, multicentre, case control study found that participants in the highest quintile of flavonoid intake, in particular flavanols and flavonols, had a 10% lower risk of developing t2D than those in the lowest quintile [63]. A meta-analysis of prospective studies of consumption of apples and pears, high in flavanols, found an inverse association between intake and t2D [64]. The long-term effects of coffee consumption are convincingly linked to reductions in t2D development [65,66] and decreases in IR [67], and a meta-analysis on epidemiological studies on anthocyanin-rich foods concluded that a higher intake of anthocyanins improved HOMA-IR through changes in fasting insulin [68].

### 3.4. Interventions Using Foods 

#### 3.4.1. Cocoa and (−)-Epicatechin

The consumption of cocoa flavanols, such as (−)-epicatechin, improves insulin sensitivity in both healthy and hypertensive populations. When comparing white and dark chocolate consumption over 15 days, HOMA-IR was significantly lower and QUICKI was significantly higher after the latter [69,70]. In elderly subjects with mild cognitive decline, 2 months of a drink containing flavanols improved IR [71], but consumption for 5 days in obese adults did not change fasting glucose or insulin [72]. In a systematic review and meta-analysis of randomised, controlled trials, it was concluded that flavanol-rich cocoa consumption decreased HOMA-IR [73]. In healthy adults consuming pure (−)-epicatechin for 1 month, HOMA-IR was improved through changes in fasting insulin with no change in fasting glucose [74].

#### 3.4.2. Green Tea

Green tea contains high amounts of galloylated catechins, such as (−)-epigallocatechin gallate and (−)-epicatechin gallate. Data on green tea consumption and IR are not entirely consistent. In one meta-analysis on green tea, there was no change in IR and glycaemic control in t2D patients [75], whereas other meta-analyses have demonstrated improvements in fasting plasma blood glucose when green tea is consumed for 0.5 to 2 months [76,77].

#### 3.4.3. Anthocyanins and Berries

Although conclusions from studies on anthocyanins are mixed, in general it appears that anthocyanins and anthocyanin-rich foods improve IR. In a systematic review of 19 randomised controlled trials, anthocyanin supplementation improved HOMA-IR [78], and three out of six studies showed a positive effect on glycaemic profile with consumption of berries [79]. Consumption of blueberry extracts, rich in anthocyanins and other (poly)phenols, improved HOMA-IR in patients with t2D but not in insulin resistant adults, and blueberry consumption improved IR in obese and insulin-resistant adults in one study but not in two other studies (summarised in [80]). In overweight and obese subjects, the consumption of proanthocyanidins and anthocyanins for 2 months prevented increases in IR induced by a fructose challenge [81]. In diabetic patients, consumption of anthocyanins for 6 months also reduced IR [82].

#### 3.4.4. Quercetin and Onions

The effect of quercetin or quercetin-rich foods on IR in unclear owing to a limited number of studies. A systematic review concluded that quercetin for 2 months reduced fasting plasma glucose, but only at high doses [83]. Supplementation with quercetin-3-*O*-glucoside for 1 month to prehypertensive but otherwise healthy men showed no effect on IR [74], and supplementation with 500 mg quercetin for 1 month did not change fasting glucose in mildly hyperuricaemic men [84]. In breast cancer patients, yellow onions containing quercetin for 2 months resulted in a decrease in blood insulin and QUICKI, but this did not translate to a change in HOMA-IR nor HOMA-β [85]. It is notable that a systematic review on 13 animal studies concluded that quercetin dose-dependently lowered fasting blood glucose [86].

#### 3.4.5. Phenolic Acids and Other Phenolic Compounds

The effect of consumption of coffee containing phenolic acids on IR is controversial, with studies reporting opposing conclusions. A trial for 8 weeks of up to 5 cups of coffee per day found no changes in insulin or glucose markers [87]. In a systematic review including six studies, green coffee extract lowered fasting blood glucose, but only higher doses were effective at improving HOMA-IR [88]. Phenolic compounds found in olive leaf, oleuropein and hydroxytyrosol, improved both the action of insulin and secretion from pancreatic β-cells following a 12-week supplementation in middle aged overweight men [89].

#### 3.4.6. Stilbenes and Wine

Consumption of resveratrol and wine appear to have limited effects on IR, but the results are contradictory even in systematic reviews addressing similar criteria. Supplementation with the stilbene, resveratrol, unique to grapes and red wine, at much concentrations higher than in the fruit or in wine, attenuated fructose-induced oxidative stress and IR in first-degree relatives of type 2 diabetic patients and in obese individuals [81,90]. Red wine consumption for 2 weeks improved IR in people with t2D [91] but on the other hand there were no improvements in insulin sensitivity in obese subjects after 8 weeks of supplementation with (poly)phenols extracted from red wine [92]. A meta-analysis considering nine randomised intervention studies on t2D patients concluded that there was no effect of wine on fasting glucose and insulin [93], with a similar conclusion on t2D patients reached for pure resveratrol in one publication [94], but with a beneficial effect on HOMA-IR reported in an alternative systematic review on resveratrol [95]. Another systematic review concluded that resveratrol did affect fasting blood glucose levels, but only at high doses for >2.5 months [96] and in a systematic review on grape polyphenols, there was no apparent improvement of IR [97].

#### 3.4.7. Hesperidin and Citrus Fruits

According to systematic review including six trials, hesperidin supplementation had no effect on fasting glucose, insulin, QUICKI nor HOMA-IR, despite positive indications from animal studies [98].

#### 3.4.8. Pomegranate and Ellagitannins

In a systematic review, pomegranate intake did not show any improvements in glucose and insulin metabolism in healthy or compromised individuals [99], nor in patients with t2D [100].

#### 3.4.9. (Poly)phenols from Nuts

Nuts contain a high quantity of fats, certain types of which may contribute to improving IR. In addition, nuts contain (poly)phenols, which could contribute to their ability to affect metabolic status. Pecans are particularly high in proanthocyanidins and ellagitannins, pistachios and almonds are high in proanthocyanidins, and walnuts are high in ellagitannins [101]. Consumption of pistachio nuts, according to 6 intervention studies, improved HOMA-IR through changes in fasting glucose [102], but a systematic review on walnuts concluded that there was no effect on fasting blood glucose or other measures of IR [103]. Although there are fewer reported studies on humans, pecan and almond consumption seem to improve HOMA-IR [104,105] and almonds are also effective at reducing IR in t2D patients [106].

## 4. Mechanisms in the Pathway of Developing IR That May Be Affected by (Poly)phenols

In assessing the mechanism of (poly)phenols on insulin signalling and resistance, considerations need to be given to the use of the appropriate model, the physiological concentration of (poly)phenols in the right form, and the biochemical markers measured. Numerous publications report cell signalling changes after treatment with a high concentration of (poly)phenol, far above that which is physiologically relevant. It is also necessary to consider data from human interventions, and if no evidence of a beneficial effect is available, then it seems irrelevant to study mechanisms in vitro. Based on this reasoning, (−)-epicatechin is one of the best candidate (poly)phenols to affect IR. Data on quercetin in humans is quite limited. Resveratrol and hesperidin do not seem to affect IR in human interventions, but phenolic acids could be active. Anthocyanins seem effective in vivo in some studies, but since anthocyanins are poorly bioavailable, then it is most likely that the effects are due to anthocyanin catabolites, not the parent molecules. Even though some (poly)phenols are not active on IR as measured by fasting blood glucose and insulin, this does not mean that they do not affect metabolic disease risk. For example, there may be effects on blood pressure, cholesterol, uric acid or triglycerides, which would also affect cardiovascular disease and t2D risk.

There are some indications that lack of insulin responsiveness is temporary, and is reversible under the right circumstances [107]. IR transiently results from a constant accelerated formation of ROS in tissues, caused by obesity/chronic inflammation and high glucose/NADPH oxidase (NOX) in muscle and adipose. In the short term, the ROS are not enough to destroy the adipose or muscle cells, but put the metabolism off balance with impaired response to insulin owing to pathway disruption. However, ultimately the ROS stress will lead to the recruitment of pro-inflammatory cells, eventually causing permanent damage [108]. In response to the lack of insulin responsiveness, β-cells produce more and more insulin to try to elicit the clearance of glucose. This over-activity of the β-cells causes permanent damage and burnout, exacerbated by the presence of pro-inflammatory cytokines, in a highly complex mechanism [109]. Pancreatic β-cells have low levels of glutathione peroxidase and almost no catalase, and so are highly sensitive to ROS. Excessive prolonged ROS production can readily cause oxidative stress and lead to a deterioration of β-cell function [110]. Since the initial stages of insulin responsiveness are reversible, then there are several mechanisms by which (poly)phenols can improve the condition, which are briefly summarised below. In addition, the models used and mechanisms involved in protecting the β-cells from damage are summarised in Section 5 below.

### 4.1. Effects of (Poly)phenols on Starch Digestion

**Figure 1:**
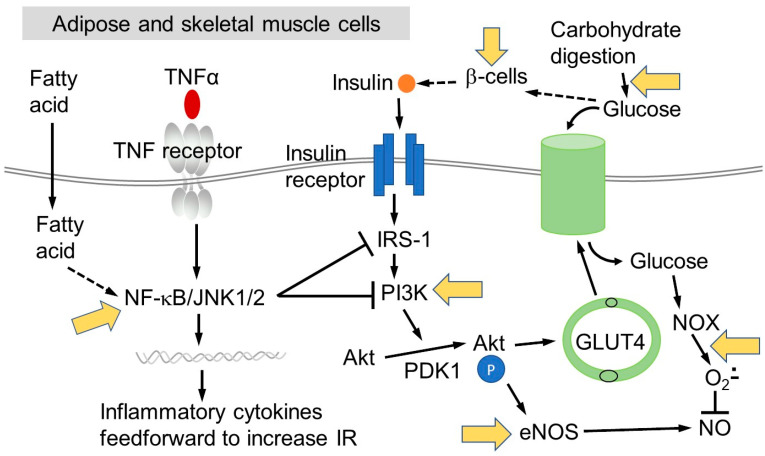
Simplified mechanism of glucose uptake by adipose or muscle tissue. Data combined from: [4,10,24,47,111,130]. Abbreviations defined in text, except for: JNK1/2, c-Jun N-terminal kinase 1/2; PDK1, pyruvate dehydrogenase kinase 1; IRS-1, insulin receptor substrate 1; PI3K, phosphoinositide 3-kinase; eNOS, endothelial nitric oxide synthase.

We and other have published reviews on the effects of (poly)phenols on postprandial glucose spikes through inhibition of carbohydrate digestion by enzyme inhibition [111,112] or by carbohydrate binding [113]. Starch is digested in the gut by α-amylases and α-glucosidases to produce glucose, which is then absorbed into the circulation. Glucose and fructose from dietary sucrose are also rapidly absorbed after hydrolysis by sucrase on the brush border of the small intestine enterocyte [114]. A high-carbohydrate meal consisting of starch and/or sugar will lead to a rapid increase of blood glucose, causing a release of insulin from β-cells to allow clearance of the glucose into tissues via GLUT4 activation by translocation [115]. Certain (poly)phenols can inhibit α-amylases and α-glucosidases and so slow down the rate of digestion, blunting glucose spikes. Since rapidly elevated glucose leads to formation of ROS in endothelial cells via NADPH oxidase 4 (NOX4) [116,117], then a smaller rise in glucose will give lower intracellular ROS in insulin-responsive cells. This leads to greater insulin sensitivity owing to less disruption of the pathways shown in Figure 1. The key requirements here are that the inhibition of α-amylase and α-glucosidase is strong enough to have an effect on blood glucose postprandially. Acarbose is a drug used to manage diabetes and is a potent non-absorbed inhibitor of α-amylase and α-glucosidase, and long-term use leads to lowered risk of t2D [118]. To judge effectiveness, (poly)phenols can be compared to acarbose. Since (poly)phenols are consumed normally and continually in a habitual plant-rich diet, they do not have to be as potent as acarbose, but they must be sufficiently effective. Only a limited number of (poly)phenols are potent enough inhibitors to be effective in vivo, even though there have hundreds of publications on this activity. The most promising and potent (poly)phenols are punicalagin [119], epigallocatechin gallate [120,121] and quercetagetin [122], although the most pronounced effects are from foods [123], implying some interaction or synergy between (poly)phenols and other food components such as fibre.

### 4.2. Effects of (Poly)phenols on Glucose Transport

The inhibition by (poly)phenols on glucose transport is also a potential mechanism to lower postprandial blood glucose [124,125]. In order to exert a positive effect on insulin sensitivity and lower blood glucose after a meal, it would be necessary to attenuate glucose transporters in the gut to slow down absorption, but to stimulate glucose transport into tissues to exert an insulin-like effect. Clearly, it is unrealistic to expect the same compound to inhibit a transporter at one site and stimulate at another. There is some evidence that intact parent (poly)phenols, such as quercetin and green tea catechins can inhibit glucose uptake in the gut [126,127], and that (poly)phenol gut microbial metabolites could stimulate glucose uptake in tissues by through glucose transporter GLUT4- and PI3K-dependent mechanisms [128]. Further, quercetin increased GLUT4 translocation and akt signalling in mouse epididymis adipose tissues [129]. This dual effect of parent compounds and microbial products is a promising line of exploration and could conceivably lead to synergistic effects between the parent compound and its microbial degradation products.

### 4.3. Attenuation of Impaired Insulin Signalling and Pro-Inflammatory Pathways by (Poly)phenols

(Poly)phenols can have multiple potential effects on the pathways by which cells take up glucose in response to insulin (Figure 1). Mechanistic information has been mostly derived from cultured cells or animal models. Since any action on non-intestinal cells is mediated through the blood, then the active compound must teach the target tissue in sufficient concentration and in an active form [131]. There are many examples in the literature where cells have been treated with (poly)phenols and some molecular players in the insulin responsive pathway have been modulated. Here, only a selection are reported to illustrate the types of studies that have been done [132], where the action was observed at a reasonable concentration and the studies shown are certainly not exhaustive. In differentiated white adipocyte 3T3-L1 cells, (−)-epicatechin at relatively low concentrations dose-dependently inhibited the effects of tumour necrosis factor (TNF)-α-induced activation of mitogen activated protein kinase (MAPK), NF-kB, and AP-1 [133]. In adipocytes under inflammatory conditions where IR is impaired, quercetin restored GLUT4 translocation by beneficial regulation of IRS-1 function [134]. In rats given high fructose or mice given a high fat diet, (−)-epicatechin attenuated the negative effects of the dietary stress through NOX4, NF-kB, and extracellular signal-regulated protein kinase (ERK1/2) pathway modulation [135,136]. Ferulic acid dose-dependently improved HOMA-IR in rats fed a high fat, high carbohydrate and high fructose diet, at least partly through downregulation of NOX and inhibition of inflammatory cytokine production [137].

## 5. Cell Models for Protection of β-Cells against Oxidative Damage

Numerous insulin-secreting cell lines are used to understand more about the mechanism of developing IR. A majority of these release insulin in response to glucose, and have been obtained from rats, mice or humans. A summary is shown in Table 1. Of these, INS-1 cells obtained from rat pancreatic β-cells have been utilised in numerous studies with (poly)phenols. Other cells are also used to examine the mechanism of modulation of IR in tissues, including adipose and muscle tissue-derived cells. 

Quercetin can protect INS-1 β-cells under stress conditions, potentiating glucose-induced insulin secretion [138], through activation of ERK1/2 [139]. At lower concentrations of glucose, quercetin can also stimulate insulin secretion via Ca^2+^ influx [140]. Under fructose-induced hyperinsulinaemia, quercetin can reduce insulin production by INS-1 β-cells and this correlates with decreased nuclear Foxo1 localization and reduced phosphorylation of akt [141]. (−)-Epicatechin at low concentration can improve glucose-stimulated insulin secretion in INS-1 cells impaired by saturated fatty acids [142], and at high concentrations, can reduce the production of glucose-induced ROS [142]. (−)-Epicatechin can also improve INS-1 cell viability and insulin secretion under chemically induced oxidative stress [143]. (−)-Epigallocatechin-3-gallate protects INS-1 cells against ethanol-induced apoptosis via the upregulation of the transcription factor NeuroD1, resulting in a decreased CCAAT-enhancer-binding protein (C/EBP) homologous protein transcription factor expression and decreased apoptosis [144]. Anthocyanins promote insulin secretion from β-cells in the presence of glucose [145], linked to intracellular Ca^2+^ signalling in insulin secretion [146]. Anthocyanins have also been shown to protect INS-1 cells from autophagic cell death induced by H_2_O_2_ stimulation [147]. Finally, ferulic acid stimulated insulin secretion in INS-1 β-cells, as measured by a rise in Ca^2+^ [148].

## 6. Conclusions

There are many studies examining the effect of (poly)phenols on insulin resistance in human intervention studies, in animal models and in cells. It can be concluded that (−)-epicatechin and (−)-epicatechin-rich foods such as cocoa can improve IR, and anthocyanins are also probably active. However, for other classes, there are still too many uncertainties to conclude that there is a protective effect. In addition, the mechanisms are still not clear, and there is a need for mechanistic studies using chronic low concentrations of relevant (poly)phenols. In addition, there are vast gaps in knowledge on the effect of (poly)phenol catabolites derived from the gut microbiota on the processes leading to IR. It seems that the (poly)phenols are a highly promising class of commonly consumed dietary compounds for improving IR, but more research is needed in order to give better dietary advice and improve the prospects of metabolically at-risk groups.

## Figures and Tables

**Table 1:** Cell models used to study effects on pancreatic β-cells.

Name	Organism	Characteristics	Studies and Reference
INS-1	Rat	β-cell line secreting insulin in response to glucose. Line is stable with time, with high glucose-induced insulin secretion	Polysaccharide-induced insulin secretion [149] Lipotoxicity [150] Bacteria [151] Cytotoxic effects of mangostin [152] Glycogen metabolism [153] Effect of Indian culinary plants [154]
RIN	Rat	Exhibits glucose-stimulated insulin secretion	[155,156,157]
Min6	Mouse	Releases insulin in response to glucose, but high passage cells have impaired insulin secretion	[158] Establishment [159] Ginseng root-treated [160] Statin-treated [161] [162]
1.1B4	Human	Secretes low levels of insulin when glucose-stimulated	Cellular response to hyperglycaemia [163] [164,165,166,167]
EndoCβH1	Human	Secretes insulin in response to a glucose challenge, but slow growing and difficult to culture	Characterisation [168] Validity in drug screening against mouse model [169] [170,171] Further studies listed in review [172]

## References

[1] Moller D.E., Kaufman K.D. (2005). Metabolic Syndrome: A Clinical and Molecular Perspective. Annu. Rev. Med..

[2] Lumeng C.N.-K., Saltiel A.R. (2011). Inflammatory links between obesity and metabolic disease. J. Clin. Investig..

[3] Gregor M.F., Hotamisligil G.S. (2011). Inflammatory Mechanisms in Obesity. Annu. Rev. Immunol..

[4] Samuel V.T., Shulman G.I. (2012). Mechanisms for Insulin Resistance: Common Threads and Missing Links. Cell.

[5] Kau A.L., Ahern P.P., Griffin N.W., Goodman A.L., Gordon J.I. (2011). Human nutrition, the gut microbiome and the immune system. Nature.

[6] Nicholson J.K., Holmes E., Kinross J., Burcelin R., Gibson G., Jia W., Pettersson S. (2012). Host-Gut Microbiota Metabolic Interactions. Science.

[7] Johnson A.M., Olefsky J.M. (2013). The Origins and Drivers of Insulin Resistance. Cell.

[8] Czech M.P. (2017). Insulin action and resistance in obesity and type 2 diabetes. Nat. Med..

[9] Fonseca V.A. (2009). Defining and Characterizing the Progression of Type 2 Diabetes. Diabetes Care.

[10] Cusi K., Maezono K., Osman A., Pendergrass M., Patti M.E., Pratipanawatr T., DeFronzo R.A., Kahn C.R., Mandarino L.J. (2000). Insulin resistance differentially affects the PI 3-kinase– and MAP kinase–mediated signaling in human muscle. J. Clin. Investig..

[11] Ishiki M., Klip A. (2005). Minireview: Recent Developments in the Regulation of Glucose Transporter-4 Traffic: New Signals, Locations, and Partners. Endocrinology.

[12] DeFronzo R.A., Tobin J.D., Andres R. (1979). Glucose clamp technique: A method for quantifying insulin secretion and resistance. Am. J. Physiol..

[13] Matthews D.R., Hosker J.P., Rudenski A.S., Naylor B.A., Treacher D.F., Turner R.C. (1985). Homeostasis model assessment: Insulin resistance and ?-cell function from fasting plasma glucose and insulin concentrations in man. Diabetologia.

[14] Rudenski A.S., Matthews D.R., Levy J.C., Turner R.C. (1991). Understanding “insulin resistance”: Both glucose resistance and insulin resistance are required to model human diabetes. Metabolism.

[15] Chen H., Sullivan G., Yue L.Q., Katz A., Quon M.J. (2003). QUICKI is a useful index of insulin sensitivity in subjects with hypertension. Am. J. Physiol. Endocrinol. Metab..

[16] Matsuda M., DeFronzo R.A. (1999). Insulin sensitivity indices obtained from oral glucose tolerance testing: Comparison with the euglycemic insulin clamp. Diabetes Care.

[17] Szosland K., Lewiński A. (2016). In quest for method of insulin resistance assessment in everyday clinical practice—Insulin resistance indices. Diabetes Metab. Syndr. Clin. Res. Rev..

[18] Simental-Mendía L.E., Rodríguez-Morán M., Guerrero-Romero F. (2008). The Product of Fasting Glucose and Triglycerides as Surrogate for Identifying Insulin Resistance in Apparently Healthy Subjects. Metab. Syndr. Relat. Disord..

[19] Borai A., Livingstone C., Kaddam I., Ferns G. (2011). Selection of the appropriate method for the assessment of insulin resistance. BMC Med. Res. Methodol..

[20] Bonora E., Kiechl S., Willeit J., Oberhollenzer F., Egger G., Meigs J.B., Bonadonna R.C., Muggeo M. (2007). Insulin Resistance as Estimated by Homeostasis Model Assessment Predicts Incident Symptomatic Cardiovascular Disease in Caucasian Subjects From the General Population: The Bruneck Study. Diabetes Care.

[21] Hanley A.J., Williams K., Stern M.P., Haffner S.M. (2002). Homeostasis Model Assessment of Insulin Resistance in Relation to the Incidence of Cardiovascular Disease: The San Antonio Heart Study. Diabetes Care.

[22] Shoelson S.E., Lee J., Yuan M. (2003). Inflammation and the IKKβ/IκB/NF-κB axis in obesity—And diet-induced insulin resistance. Int. J. Obes..

[23] Hotamisligil G.S., Shargill N.S., Spiegelman B.M. (1993). Adipose expression of tumor necrosis factor-alpha: Direct role in obesity-linked insulin resistance. Science.

[24] Hotamisligil G.K.S., Peraldi P., Budavari A., Ellis R., White M.F., Spiegelman B.M. (1996). IRS-1-Mediated Inhibition of Insulin Receptor Tyrosine Kinase Activity in TNF-alpha- and Obesity-Induced Insulin Resistance. Science.

[25] Zick Y. (2001). Insulin resistance: A phosphorylation-based uncoupling of insulin signaling. Trends Cell Biol..

[26] Arkan M.C., Hevener A.L., Greten F.R., Maeda S., Li Z.-W., Long J.M., Wynshaw-Boris A., Poli G., Olefsky J., Karin M. (2005). IKK-β links inflammation to obesity-induced insulin resistance. Nat. Med..

[27] Osborn O., Olefsky J.M. (2012). The cellular and signaling networks linking the immune system and metabolism in disease. Nat. Med..

[28] Cai D., Yuan M., Frantz D.F., Melendez P.A., Hansen L., Lee J., Shoelson S.E. (2005). Local and systemic insulin resistance resulting from hepatic activation of IKK-β and NF-κB. Nat. Med..

[29] Itani S.I., Ruderman N.B., Schmieder F., Boden G. (2002). Lipid-induced insulin resistance in human muscle is associated with changes in diacylglycerol, protein kinase C, and IkappaB-alpha. Diabetes.

[30] Bandyopadhyay G.K., Yu J.G., Ofrecio J., Olefsky J.M. (2005). Increased p85/55/50 expression and decreased phosphotidylinositol 3-kinase activity in insulin-resistant human skeletal muscle. Diabetes.

[31] Keane K.N., Cruzat V., Carlessi R., De Bittencourt P.I.H., Newsholme P. (2015). Molecular Events Linking Oxidative Stress and Inflammation to Insulin Resistance and β-Cell Dysfunction. Oxidative Med. Cell. Longev..

[32] Rave K., Roggen K., Dellweg S., Heise T., Dieck H.T. (2007). Improvement of insulin resistance after diet with a whole-grain based dietary product: Results of a randomized, controlled cross-over study in obese subjects with elevated fasting blood glucose. Br. J. Nutr..

[33] McKeown N.M., Meigs J.B., Liu S., Saltzman E., Wilson P.W., Jacques P.F. (2004). Carbohydrate nutrition, insulin resistance, and the prevalence of the metabolic syndrome in the Framingham Offspring Cohort. Diabetes Care.

[34] Liese A.D., Roach A.K., Sparks K.C., Marquart L., D’Agostino R.B., Mayer-Davis E.J. (2003). Whole-grain intake and insulin sensitivity: The Insulin Resistance Atherosclerosis Study. Am. J. Clin. Nutr..

[35] Jang Y., Lee J.H., Kim O.Y., Park H.Y., Lee S.Y. (2001). Consumption of whole grain and legume powder reduces insulin demand, lipid peroxidation, and plasma homocysteine concentrations in patients with coronary artery disease: Randomized controlled clinical trial. Arter. Thromb. Vasc. Biol..

[36] Pereira M.A., Jacobs D.R., Pins J.J., Raatz S.K., Gross M.D., Slavin J.L., Seaquist E.R. (2002). Effect of whole grains on insulin sensitivity in overweight hyperinsulinemic adults. Am. J. Clin. Nutr..

[37] Frost G., Leeds A., Trew G., Margara R., Dornhorst A. (1998). Insulin sensitivity in women at risk of coronary heart disease and the effect of a low glycemic diet. Metabolism.

[38] Paniagua J.A., De La Sacristana A.G., Romero I., Vidal-Puig A., Latre J., Sánchez E., Perez-Jimenez F., López-Miranda J., Perez-Jimenez F. (2007). Monounsaturated Fat-Rich Diet Prevents Central Body Fat Distribution and Decreases Postprandial Adiponectin Expression Induced by a Carbohydrate-Rich Diet in Insulin-Resistant Subjects. Diabetes Care.

[39] Ramel A., Martinez A., Kiely M., Morais G., Bandarra N.M., Thorsdottir I. (2008). Beneficial effects of long-chain n-3 fatty acids included in an energy-restricted diet on insulin resistance in overweight and obese European young adults. Diabetologia.

[40] Hartweg J., Perera R., Montori V.M., Dinneen S.F., Neil A.H., Farmer A., Neil H.A.W. (2008). Omega-3 polyunsaturated fatty acids (PUFA) for type 2 diabetes mellitus. Cochrane Database Syst. Rev..

[41] Bachmann O.P., Dahl D.B., Brechtel K., Machann J., Haap M., Maier T., Loviscach M., Stumvoll M., Claussen C.D., Schick F. (2001). Effects of Intravenous and Dietary Lipid Challenge on Intramyocellular Lipid Content and the Relation With Insulin Sensitivity in Humans. Diabetes.

[42] Pehleman T.L., Peters S.J., Heigenhauser G.J.F., Spriet L.L. (2005). Enzymatic regulation of glucose disposal in human skeletal muscle after a high-fat, low-carbohydrate diet. J. Appl. Physiol..

[43] Bisschop P.H., De Metz J., Ackermans M.T., Endert E., Pijl H., Kuipers F., Meijer A.J., Sauerwein H.P., Romijn J.A. (2001). Dietary fat content alters insulin-mediated glucose metabolism in healthy men. Am. J. Clin. Nutr..

[44] Brøns C., Jensen C.B., Storgaard H., Hiscock N.J., White A., Appel J.S., Jacobsen S., Nilsson E., Larsen C.M., Astrup A. (2009). Impact of short-term high-fat feeding on glucose and insulin metabolism in young healthy men. J. Physiol..

[45] Von Frankenberg A.D., Marina A., Song X., Callahan H.S., Kratz M., Utzschneider K.M. (2015). A high-fat, high-saturated fat diet decreases insulin sensitivity without changing intra-abdominal fat in weight-stable overweight and obese adults. Eur. J. Nutr..

[46] Bakker L.E.H., Van Schinkel L.D., Guigas B., Streefland T.C.M., Jonker J.T., Van Klinken J.B., Van Der Zon G.C.M., Lamb H.J., Smit J.W., Pijl H. (2014). A 5-Day High-Fat, High-Calorie Diet Impairs Insulin Sensitivity in Healthy, Young South Asian Men but Not in Caucasian Men. Diabetes.

[47] Glass C.K., Olefsky J.M. (2012). Inflammation and Lipid Signaling in the Etiology of Insulin Resistance. Cell Metab..

[48] Fabbrini E., Magkos F., Mohammed B.S., Pietka T., Abumrad N.A., Patterson B.W., Okunade A., Klein S. (2009). Intrahepatic fat, not visceral fat, is linked with metabolic complications of obesity. Proc. Natl. Acad. Sci. USA.

[49] Fabbrini E., Tamboli R.A., Magkos F., Marks–Shulman P.A., Eckhauser A.W., Richards W.O., Klein S., Abumrad N.N. (2010). Surgical Removal of Omental Fat Does Not Improve Insulin Sensitivity and Cardiovascular Risk Factors in Obese Adults. Gastroenterology.

[50] Cani P.D., Amar J., Iglesias M.A., Poggi M., Knauf C., Bastelica D., Neyrinck A.M., Fava F., Tuohy K.M., Chabo C. (2007). Metabolic Endotoxemia Initiates Obesity and Insulin Resistance. Diabetes.

[51] Kumar A.S., Maiya G.A., Shastry B., Vaishali K., Ravishankar N., Hazari A., Gundmi S., Jadhav R. (2019). Exercise and insulin resistance in type 2 diabetes mellitus: A systematic review and meta-analysis. Ann. Phys. Rehabil. Med..

[52] Marson E.C., Delevatti R.S., Prado A.K.G., Netto N., Kruel L.F.M. (2016). Effects of aerobic, resistance, and combined exercise training on insulin resistance markers in overweight or obese children and adolescents: A systematic review and meta-analysis. Prev. Med..

[53] Kay C.D., Clifford M.N., Mena P., McDougall G.J., Andres-Lacueva C., Cassidy A., Del Rio D., Kuhnert N., Manach C., Pereira-Caro G. (2020). Recommendations for standardizing nomenclature for dietary (poly)phenol catabolites. Am. J. Clin. Nutr..

[54] Frank J., Fukagawa N.K., Bilia A.R., Johnson E.J., Kwon O., Prakash V., Miyazawa T., Clifford M.N., Kay C.D., Crozier A. (2019). Terms and nomenclature used for plant-derived components in nutrition and related research: Efforts toward harmonization. Nutr. Rev..

[55] Del Rio D., Rodriguez-Mateos A., Spencer J.P., Tognolini M., Borges G., Crozier A. (2013). Dietary (Poly)phenolics in Human Health: Structures, Bioavailability, and Evidence of Protective Effects Against Chronic Diseases. Antioxid. Redox Signal..

[56] Clifford M.N., Van Der Hooft J.J., Crozier A. (2013). Human studies on the absorption, distribution, metabolism, and excretion of tea polyphenols. Am. J. Clin. Nutr..

[57] Neveu V., Perez-Jimenez J., Vos F., Crespy V., Du Chaffaut L., Mennen L., Knox C., Eisner R., Cruz J., Wishart D. (2010). Phenol-Explorer: An online comprehensive database on polyphenol contents in foods. Database.

[58] Williamson G., Kay C.D., Crozier A. (2018). The Bioavailability, Transport, and Bioactivity of Dietary Flavonoids: A Review from a Historical Perspective. Compr. Rev. Food Sci. Food Saf..

[59] Actis-Goretta L., Lévèques A., Giuffrida F., Romanov-Michailidis F., Viton F., Barron D., Duenas-Paton M., Gonzalez-Manzano S., Santos-Buelga C., Williamson G. (2012). Elucidation of (−)-epicatechin metabolites after ingestion of chocolate by healthy humans. Free Radic. Biol. Med..

[60] Almeida A.F., Borge G.I.A., Piskula M.K., Tudose A., Tudoreanu L., Valentová K., Williamson G., Dos Santos C.N. (2018). Bioavailability of Quercetin in Humans with a Focus on Interindividual Variation. Compr. Rev. Food Sci. Food Saf..

[61] Clifford M.N., Kerimi A., Williamson G. (2020). Bioavailability and metabolism of chlorogenic acids (acyl-quinic acids) in humans. Compr. Rev. Food Sci. Food Saf..

[62] Maghsoudi Z., Ghiasvand R., Salehi-Abargouei A. (2015). Empirically derived dietary patterns and incident type 2 diabetes mellitus: A systematic review and meta-analysis on prospective observational studies. Public Health Nutr..

[63] Zamora-Ros R., Forouhi N.G., Sharp S.J., González C.A., Buijsse B., Guevara M., Van Der Schouw Y.T., Amiano P., Boeing H., Bredsdorff L. (2013). The Association Between Dietary Flavonoid and Lignan Intakes and Incident Type 2 Diabetes in European Populations: The EPIC-InterAct study. Diabetes Care.

[64] Guo X.-F., Yang B., Tang J., Jiang J.-J., Li D. (2017). Apple and pear consumption and type 2 diabetes mellitus risk: A meta-analysis of prospective cohort studies. Food Funct..

[65] Bhupathiraju S.N., Pan A., Malik V.S., Manson J.E., Willett W.C., Van Dam R.M., Hu F.B. (2012). Caffeinated and caffeine-free beverages and risk of type 2 diabetes. Am. J. Clin. Nutr..

[66] Ding M., Bhupathiraju S.N., Chen M., van Dam R.M., Hu F.B. (2014). Caffeinated and Decaffeinated Coffee Consumption and Risk of Type 2 Diabetes: A Systematic Review and a Dose-Response Meta-analysis. Diabetes Care.

[67] Lee C.B., Yu S.H., Kim N.Y., Kim S.M., Kim S.R., Oh S.J., Jee S.H., Lee J.E. (2017). Association Between Coffee Consumption and Circulating Levels of Adiponectin and Leptin. J. Med. Food.

[68] Jennings A., Welch A.A., Spector T., MacGregor A., Cassidy A. (2013). Intakes of Anthocyanins and Flavones Are Associated with Biomarkers of Insulin Resistance and Inflammation in Women. J. Nutr..

[69] Grassi D., Lippi C., Necozione S., Desideri G., Ferri C. (2005). Short-term administration of dark chocolate is followed by a significant increase in insulin sensitivity and a decrease in blood pressure in healthy persons. Am. J. Clin. Nutr..

[70] Grassi D., Desideri G., Necozione S., Lippi C., Casale R., Properzi G., Blumberg J.B., Ferri C. (2008). Blood Pressure Is Reduced and Insulin Sensitivity Increased in Glucose-Intolerant, Hypertensive Subjects after 15 Days of Consuming High-Polyphenol Dark Chocolate. J. Nutr..

[71] Desideri G., Kwik-Uribe C., Grassi D., Necozione S., Ghiadoni L., Mastroiacovo D., Raffaele A., Ferri L., Bocale R., Lechiara M.C. (2012). Benefits in Cognitive Function, Blood Pressure, and Insulin Resistance Through Cocoa Flavanol Consumption in Elderly Subjects With Mild Cognitive Impairment. Hypertension.

[72] Stote K.S., Clevidence B.A., Novotny J.A., Henderson T., Radecki S.V., Baer D.J. (2012). Effect of cocoa and green tea on biomarkers of glucose regulation, oxidative stress, inflammation and hemostasis in obese adults at risk for insulin resistance. Eur. J. Clin. Nutr..

[73] Shrime M.G., Bauer S.R., McDonald A.C., Chowdhury N.H., Coltart C.E.M., Ding E.L. (2011). Flavonoid-Rich Cocoa Consumption Affects Multiple Cardiovascular Risk Factors in a Meta-Analysis of Short-Term Studies. J. Nutr..

[74] Dower J.I., Geleijnse J.M., Gijsbers L., Zock P.L., Kromhout D., Hollman P.C.H. (2015). Effects of the pure flavonoids epicatechin and quercetin on vascular function and cardiometabolic health: A randomized, double-blind, placebo-controlled, crossover trial. Am. J. Clin. Nutr..

[75] Wang X., Tian J., Jiang J., Li L., Ying X., Tian H., Nie M. (2013). Effects of green tea or green tea extract on insulin sensitivity and glycaemic control in populations at risk of type 2 diabetes mellitus: A systematic review and meta-analysis of randomised controlled trials. J. Hum. Nutr. Diet..

[76] Liu K., Zhou R., Wang B., Chen K., Shi L.-Y., Zhu J.-D., Mi M.-T. (2013). Effect of green tea on glucose control and insulin sensitivity: A meta-analysis of 17 randomized controlled trials. Am. J. Clin. Nutr..

[77] Zheng X.-X., Xu Y.-L., Li S.-H., Hui R., Wu Y., Huang X.-H. (2013). Effects of green tea catechins with or without caffeine on glycemic control in adults: A meta-analysis of randomized controlled trials. Am. J. Clin. Nutr..

[78] Daneshzad E., Shab-Bidar S., Mohammadpour Z., Djafarian K. (2019). Effect of anthocyanin supplementation on cardio-metabolic biomarkers: A systematic review and meta-analysis of randomized controlled trials. Clin. Nutr..

[79] Heneghan C., Kiely M., Lyons J., Lucey A. (2018). The Effect of Berry-Based Food Interventions on Markers of Cardiovascular and Metabolic Health: A Systematic Review of Randomized Controlled Trials. Mol. Nutr. Food Res..

[80] Stull A.J., Cash K.C., Johnson W.D., Champagne C.M., Cefalu W.T. (2010). Bioactives in Blueberries Improve Insulin Sensitivity in Obese, Insulin-Resistant Men and Women. J. Nutr..

[81] Hokayem M., Blond E., Vidal H., Lambert K., Meugnier E., Feillet-Coudray C., Coudray C., Pesenti S., Luyton C., Lambert-Porcheron S. (2013). Grape Polyphenols Prevent Fructose-Induced Oxidative Stress and Insulin Resistance in First-Degree Relatives of Type 2 Diabetic Patients. Diabetes Care.

[82] Liu Y., Li D., Zhang Y., Sun R., Xia M. (2014). Anthocyanin increases adiponectin secretion and protects against diabetes-related endothelial dysfunction. Am. J. Physiol. Endocrinol. Metab..

[83] Ostadmohammadi V., Milajerdi A., Ayati E., Kolahdooz F., Asemi Z. (2019). Effects of quercetin supplementation on glycemic control among patients with metabolic syndrome and related disorders: A systematic review and meta-analysis of randomized controlled trials. Phytother. Res..

[84] Shi Y., Williamson G. (2016). Quercetin lowers plasma uric acid in pre-hyperuricaemic males: A randomised, double-blinded, placebo-controlled, cross-over trial. Br. J. Nutr..

[85] Jafarpour-Sadegh F., Montazeri V., Adili A., Esfehani A., Rashidi M.-R., Pirouzpanah S. (2016). Consumption of Fresh Yellow Onion Ameliorates Hyperglycemia and Insulin Resistance in Breast Cancer Patients During Doxorubicin-Based Chemotherapy: A Randomized Controlled Clinical Trial. Integr. Cancer Ther..

[86] Bule M., Abdurahman A., Nikfar S., Abdollahi M., Amini M. (2019). Antidiabetic effect of quercetin: A systematic review and meta-analysis of animal studies. Food Chem. Toxicol..

[87] Shaposhnikov S., Hatzold T., El Yamani N., Stavro P.M., Lorenzo Y., Dusinska M., Reus A., Pasman W., Collins A. (2016). Coffee and oxidative stress: A human intervention study. Eur. J. Nutr..

[88] Nikpayam O., Najafi M., Ghaffari S., Jafarabadi M.A., Sohrab G., Roshanravan N. (2019). Effects of green coffee extract on fasting blood glucose, insulin concentration and homeostatic model assessment of insulin resistance (HOMA-IR): A systematic review and meta-analysis of interventional studies. Diabetol. Metab. Syndr..

[89] De Bock M., Derraik J.G.B., Brennan C.M., Biggs J.B., Morgan P.E., Hodgkinson S.C., Hofman P.L., Cutfield W.S. (2013). Olive (*Olea europaea* L.) Leaf Polyphenols Improve Insulin Sensitivity in Middle-Aged Overweight Men: A Randomized, Placebo-Controlled, Crossover Trial. PLoS ONE.

[90] Timmers S., Konings E., Bilet L., Houtkooper R.H., Van De Weijer T., Goossens G.H., Hoeks J., Van Der Krieken S., Ryu D., Kersten S. (2011). Calorie Restriction-like Effects of 30 Days of Resveratrol Supplementation on Energy Metabolism and Metabolic Profile in Obese Humans. Cell Metab..

[91] Napoli R., Cozzolino D., Guardasole V., Angelini V., Zarra E., Matarazzo M., Cittadini A., Sacca L., Torella R. (2005). Red wine consumption improves insulin resistance but not endothelial function in type 2 diabetic patients. Metabolism.

[92] Woerdeman J., Del Rio D., Calani L., Eringa E.C., Smulders Y.M., Serné E.H. (2017). Red wine polyphenols do not improve obesity-associated insulin resistance: A randomized controlled trial. Diabetes Obes. Metab..

[93] Ye J., Chen X., Bao L. (2019). Effects of wine on blood pressure, glucose parameters, and lipid profile in type 2 diabetes mellitus. Medicine.

[94] Hausenblas H.A., Schoulda J.A., Smoliga J.M. (2015). Resveratrol treatment as an adjunct to pharmacological management in type 2 diabetes mellitus-systematic review and meta-analysis. Mol. Nutr. Food Res..

[95] Zhu X., Wu C., Qiu S., Yuan X., Li L. (2017). Effects of resveratrol on glucose control and insulin sensitivity in subjects with type 2 diabetes: Systematic review and meta-analysis. Nutr. Metab..

[96] Asgary S., Karimi R., Momtaz S., Naseri R., Farzaei M.H. (2019). Effect of resveratrol on metabolic syndrome components: A systematic review and meta-analysis. Rev. Endocr. Metab. Disord..

[97] Woerdeman J., Van Poelgeest E., Ket J.C., Eringa E.C., Serné E.H., Smulders Y.M. (2017). Do grape polyphenols improve metabolic syndrome components? A systematic review. Eur. J. Clin. Nutr..

[98] Shams-Rad S., Mohammadi M., Ramezani-Jolfaie N., Zarei S., Mohsenpour M., Salehi-Abargouei A. (2020). Hesperidin supplementation has no effect on blood glucose control: A systematic review and meta-analysis of randomized controlled clinical trials. Br. J. Clin. Pharmacol..

[99] Huang H., Liao D., Chen G., Chen H., Zhu Y. (2017). Lack of efficacy of pomegranate supplementation for glucose management, insulin levels and sensitivity: Evidence from a systematic review and meta-analysis. Nutr. J..

[100] Jandari S., Hatami E., Ziaei R., Ghavami A., Yamchi A.M. (2020). The effect of pomegranate (*Punica granatum*) supplementation on metabolic status in patients with type 2 diabetes: A systematic review and meta-analysis. Complement. Ther. Med..

[101] Alasalvar C., Bolling B.W. (2015). Review of nut phytochemicals, fat-soluble bioactives, antioxidant components and health effects. Br. J. Nutr..

[102] Nowrouzi-Sohrabi P., Hassanipour S., Sisakht M., Daryabeygi-Khotbehsara R., Savardashtaki A., Fathalipour M. (2020). The effectiveness of pistachio on glycemic control and insulin sensitivity in patients with type 2 diabetes, prediabetes and metabolic syndrome: A systematic review and meta-analysis. Diabetes Metab. Syndr. Clin. Res. Rev..

[103] Neale E.P., Guan V., Tapsell L.C., Probst Y.C. (2020). Effect of walnut consumption on markers of blood glucose control: A systematic review and meta-analysis. Br. J. Nutr..

[104] McKay D.L., Eliasziw M., Chen C.-Y.O., Blumberg J.B. (2018). A Pecan-Rich Diet Improves Cardiometabolic Risk Factors in Overweight and Obese Adults: A Randomized Controlled Trial. Nutrients.

[105] Dhillon J., Thorwald M., De La Cruz N., Vu E., Asghar S.A., Kuse Q., Rios L.K.D., Ortiz R.M. (2018). Glucoregulatory and Cardiometabolic Profiles of Almond vs. Cracker Snacking for 8 Weeks in Young Adults: A Randomized Controlled Trial. Nutrients.

[106] Li S.-C., Liu Y.-H., Liu J.-F., Chang W.-H., Chen C.-M. (2011). Almond consumption improved glycemic control and lipid profiles in patients with type 2 diabetes mellitus. Metabolism.

[107] Karam J.H. (1996). Reversible Insulin Resistance in Non-Insulin-Dependent Diabetes Mellitus. Horm. Metab. Res..

[108] Wei Y., Chen K., Whaley-Connell A.T., Stump C.S., Ibdah J.A., Sowers J.R. (2008). Skeletal muscle insulin resistance: Role of inflammatory cytokines and reactive oxygen species. Am. J. Physiol. Integr. Comp. Physiol..

[109] Donath M.Y., Ehses J.A., Maedler K., Schumann D.M., Ellingsgaard H., Eppler E., Reinecke M. (2005). Mechanisms of -Cell Death in Type 2 Diabetes. Diabetes.

[110] Lenzen S., Drinkgern J., Tiedge M. (1996). Low antioxidant enzyme gene expression in pancreatic islets compared with various other mouse tissues. Free. Radic. Biol. Med..

[111] Williamson G. (2012). Possible effects of dietary polyphenols on sugar absorption and digestion. Mol. Nutr. Food Res..

[112] Xiao J., Ni X., Kai G., Chen X. (2013). A Review on Structure–Activity Relationship of Dietary Polyphenols Inhibiting α-Amylase. Crit. Rev. Food Sci. Nutr..

[113] Patel H., Royall P.G., Gaisford S., Williams G.R., Edwards C.H., Warren F.J., Flanagan B.M., Ellis P.R., Butterworth P.J. (2017). Structural and enzyme kinetic studies of retrograded starch: Inhibition of α-amylase and consequences for intestinal digestion of starch. Carbohydr. Polym..

[114] Madariaga H., Lee P.C., Heitlinger L.A., Lebenthal E. (1988). Effects of graded alpha-glucosidase inhibition on sugar absorption in vivo. Dig. Dis. Sci..

[115] Xu Y., Rubin B.R., Orme C.M., Karpikov A., Yu C., Bogan J.S., Toomre D.K. (2011). Dual-mode of insulin action controls GLUT4 vesicle exocytosis. J. Cell Biol..

[116] Jansen F., Yang X., Franklin B.S., Hoelscher M., Schmitz T., Bedorf J., Nickenig G., Werner N. (2013). High glucose condition increases NADPH oxidase activity in endothelial microparticles that promote vascular inflammation. Cardiovasc. Res..

[117] Gao L., Mann G.E. (2009). Vascular NAD(P)H oxidase activation in diabetes: A double-edged sword in redox signalling. Cardiovasc. Res..

[118] Chiasson J.-L., Josse R.G., Gomis R., Hanefeld M., Karasik A., Laakso M. (2002). Acarbose for prevention of type 2 diabetes mellitus: The STOP-NIDDM randomised trial. Lancet.

[119] Kerimi A., Nyambe-Silavwe H., Gauer J.S., Tomas-Barberan F., Williamson G. (2017). Pomegranate juice, but not an extract, confers a lower glycemic response on a high–glycemic index food: Randomized, crossover, controlled trials in healthy subjects. Am. J. Clin. Nutr..

[120] Simsek M., Quezada-Calvillo R., Ferruzzi M.G., Nichols B.L., Hamaker B.R. (2015). Dietary Phenolic Compounds Selectively Inhibit the Individual Subunits of Maltase-Glucoamylase and Sucrase-Isomaltase with the Potential of Modulating Glucose Release. J. Agric. Food Chem..

[121] Forester S.C., Gu Y., Lambert J.D. (2012). Inhibition of starch digestion by the green tea polyphenol, (−)-epigallocatechin-3-gallate. Mol. Nutr. Food Res..

[122] Piparo E.L., Scheib H., Frei N., Williamson G., Grigorov M., Chou C.J. (2008). Flavonoids for Controlling Starch Digestion: Structural Requirements for Inhibiting Human α-Amylase. J. Med. Chem..

[123] Nyambe-Silavwe H., Williamson G. (2016). Polyphenol- and fibre-rich dried fruits with green tea attenuate starch-derived postprandial blood glucose and insulin: A randomised, controlled, single-blind, cross-over intervention. Br. J. Nutr..

[124] Gauer J.S., Tumova S., Lippiat J.D., Kerimi A., Williamson G. (2018). Differential patterns of inhibition of the sugar transporters GLUT2, GLUT5 and GLUT7 by flavonoids. Biochem. Pharmacol..

[125] Martin H.-J., Kornmann F., Fuhrmann G.F. (2003). The inhibitory effects of flavonoids and antiestrogens on the Glut1 glucose transporter in human erythrocytes. Chem. Biol. Interact..

[126] Song J., Kwon O., Chen S., Daruwala R., Eck P., Park J.B., Levine M. (2002). Flavonoid Inhibition of Sodium-dependent Vitamin C Transporter 1 (SVCT1) and Glucose Transporter Isoform 2 (GLUT2), Intestinal Transporters for Vitamin C and Glucose. J. Biol. Chem..

[127] Villa-Rodriguez J.A., Aydin E., Gauer J.S., Pyner A., Williamson G., Kerimi A. (2017). Green and Chamomile Teas, but not Acarbose, Attenuate Glucose and Fructose Transport via Inhibition of GLUT2 and GLUT5. Mol. Nutr. Food Res..

[128] Houghton M.J., Kerimi A., Mouly V., Tumova S., Williamson G. (2018). Gut microbiome catabolites as novel modulators of muscle cell glucose metabolism. FASEB J..

[129] Dong J., Zhang X., Zhang L., Bian H.-X., Xu N., Bao B., Liu J. (2014). Quercetin reduces obesity-associated ATM infiltration and inflammation in mice: A mechanism including AMPKα1/SIRT1. J. Lipid Res..

[130] Dugani C.B., Klip A. (2005). Glucose transporter 4: Cycling, compartments and controversies. EMBO Rep..

[131] Kroon P.A., Clifford M.N., Crozier A., Day A.J., Donovan J.L., Manach C., Williamson G. (2004). How should we assess the effects of exposure to dietary polyphenols in vitro?. Am. J. Clin. Nutr..

[132] Fraga C.G., Oteiza P.I. (2011). Dietary flavonoids: Role of (−)-epicatechin and related procyanidins in cell signaling. Free Radic. Biol. Med..

[133] Vazquez-Prieto M.A., Bettaieb A., Haj F.G., Fraga C.G., Oteiza P.I. (2012). (−)-Epicatechin prevents TNFα-induced activation of signaling cascades involved in inflammation and insulin sensitivity in 3T3-L1 adipocytes. Arch. Biochem. Biophys..

[134] Xu Y.-Y., Wu T.-T., Zhou S.-H., Bao Y.-Y., Wang Q.-Y., Fan J., Huang Y.-P. (2014). Apigenin suppresses GLUT-1 and p-AKT expression to enhance the chemosensitivity to cisplatin of laryngeal carcinoma Hep-2 cells: An in vitro study. Int. J. Clin. Exp. Pathol..

[135] Bettaieb A., Prieto M.A.V., Lanzi C.R., Miatello R.M., Haj F.G., Fraga C.G., Oteiza P.I. (2014). (−)-Epicatechin mitigates high-fructose-associated insulin resistance by modulating redox signaling and endoplasmic reticulum stress. Free Radic. Biol. Med..

[136] Cremonini E., Wang Z., Bettaieb A., Adamo A.M., Daveri E., Mills D.A., Kalanetra K.M., Haj F.G., Karakas S., Oteiza P.I. (2018). (−)-Epicatechin protects the intestinal barrier from high fat diet-induced permeabilization: Implications for steatosis and insulin resistance. Redox Biol..

[137] Senaphan K., Kukongviriyapan U., Sangartit W., Pakdeechote P., Pannangpetch P., Prachaney P., Greenwald S.E., Kukongviriyapan V. (2015). Ferulic Acid Alleviates Changes in a Rat Model of Metabolic Syndrome Induced by High-Carbohydrate, High-Fat Diet. Nutrients.

[138] Youl E., Bardy G., Magous R., Cros G., Sejalon F., Virsolvy A., Richard S., Quignard J., Gross R., Petit P. (2010). Quercetin potentiates insulin secretion and protects INS-1 pancreatic β-cells against oxidative damage via the ERK1/2 pathway. Br. J. Pharmacol..

[139] Youl E., Magous R., Cros G., Oiry C. (2014). MAP Kinase cross talks in oxidative stress-induced impairment of insulin secretion. Involvement in the protective activity of quercetin. Fundam. Clin. Pharmacol..

[140] Bardy G., Virsolvy A., Quignard J.F., Ravier M.A., Bertrand G., Dalle S., Cros G., Magous R., Richard S., Oiry C. (2013). Quercetin induces insulin secretion by direct activation of L-type calcium channels in pancreatic beta cells. Br. J. Pharmacol..

[141] Li J.-M., Wang W., Fan C.-Y., Wang M.-X., Zhang X., Hu Q.-H., Kong L.-D. (2013). Quercetin Preservesβ-Cell Mass and Function in Fructose-Induced Hyperinsulinemia through Modulating Pancreatic Akt/FoxO1 Activation. Evid. Based Complement. Altern. Med..

[142] Yang K., Chan C.B. (2018). Epicatechin potentiation of glucose-stimulated insulin secretion in INS-1 cells is not dependent on its antioxidant activity. Acta Pharmacol. Sin..

[143] Martín M.Á., Fernández-Millán E., Ramos S., Bravo L., Goya L. (2013). Cocoa flavonoid epicatechin protects pancreatic beta cell viability and function against oxidative stress. Mol. Nutr. Food Res..

[144] Wu T., Xiang J., Shan W., Li M., Zhou W., Han X., Chen F. (2016). Epigallocatechin-3-Gallate Inhibits Ethanol-Induced Apoptosis Through Neurod1 Regulating CHOP Expression in Pancreatic β-Cells. Anat. Rec..

[145] Jayaprakasam B., Vareed S.K., Olson L.K., Nair M.G. (2005). Insulin Secretion by Bioactive Anthocyanins and Anthocyanidins Present in Fruits. J. Agric. Food Chem..

[146] Suantawee T., Elazab S.T., Hsu W.H., Yao S., Cheng H., Adisakwattana S. (2017). Cyanidin Stimulates Insulin Secretion and Pancreatic β-Cell Gene Expression through Activation of l-type Voltage-Dependent Ca^2+^ Channels. Nutrients.

[147] Zhang B., Buya M., Qin W., Sun C., Cai H., Xie Q., Xu B., Wu Y. (2013). Anthocyanins from Chinese Bayberry Extract Activate Transcription Factor Nrf2 in β Cells and Negatively Regulate Oxidative Stress-Induced Autophagy. J. Agric. Food Chem..

[148] Adisakwattana S., Moonsan P., Yibchok-Anun S. (2008). Insulin-releasing properties of a series of cinnamic acid derivatives in vitro and in vivo. J. Agric. Food Chem..

[149] Hu Q., Niu Q., Song H., Wei S., Wang S., Yao L., Li Y.-P. (2019). Polysaccharides from *Portulaca oleracea* L. regulated insulin secretion in INS-1 cells through voltage-gated Na(+) channel. Biomed. Pharmacother..

[150] Assali E.A., Shlomo D., Zeng J., Taddeo E.P., Trudeau K.M., Erion K.A., Colby A.H., Grinstaff M.W., Liesa M., Las G. (2018). Nanoparticle-mediated lysosomal reacidification restores mitochondrial turnover and function in β cells under lipotoxicity. FASEB J..

[151] Ramenzoni L.L., Zuellig R.A., Hussain A., Lehmann R., Heumann C., Attin T., Schmidlin P.R. (2018). Bacterial supernatants elevate glucose-dependent insulin secretion in rat pancreatic INS-1 line and islet β-cells via PI3K/AKT signaling. Mol. Cell. Biochem..

[152] Lee D., Kim Y.-M., Jung K., Chin Y.-W., Kang K.S. (2018). Alpha-Mangostin Improves Insulin Secretion and Protects INS-1 Cells from Streptozotocin-Induced Damage. Int. J. Mol. Sci..

[153] Andersson L.E., Nicholas L.M., Filipsson K., Sun J., Medina A., Fex M., Mulder H., Spégel P., Al-Majdoub M. (2016). Glycogen metabolism in the glucose-sensing and supply-driven beta-cell. FEBS Lett..

[154] Kaur L., Han K.-S., Bains K., Singh H. (2011). Indian culinary plants enhance glucose-induced insulin secretion and glucose consumption in INS-1 β-cells and 3T3-L1 adipocytes. Food Chem..

[155] Cai E.P., Lin J.-K. (2009). Epigallocatechin Gallate (EGCG) and Rutin Suppress the Glucotoxicity through Activating IRS2 and AMPK Signaling in Rat Pancreatic β Cells. J. Agric. Food Chem..

[156] Al-Nahdi A.M., John A., Raza H. (2018). Cytoprotective Effects of N-Acetylcysteine on Streptozotocin- Induced Oxidative Stress and Apoptosis in RIN-5F Pancreatic β-Cells. Cell. Physiol. Biochem..

[157] Chang K.-C., Hsu C.-C., Liu S.-H., Su C.-C., Yen C.-C., Lee M.-J., Chen K.-L., Ho T.-J., Hung D.-Z., Wu C.-C. (2013). Cadmium Induces Apoptosis in Pancreatic β-Cells through a Mitochondria-Dependent Pathway: The Role of Oxidative Stress-Mediated c-Jun N-Terminal Kinase Activation. PLoS ONE.

[158] Rondas D., Tomas A., Soto-Ribeiro M., Wehrle-Haller B., Halban P.A. (2011). Novel Mechanistic Link between Focal Adhesion Remodeling and Glucose-stimulated Insulin Secretion. J. Biol. Chem..

[159] Miyazaki J.-I., Earaki K., Yamato E., Ikegami H., Asano T., Shibasaki Y., Oka Y., Yamamura K.-I. (1990). Establishment of a Pancreatic β Cell Line That Retains Glucose-Inducible Insulin Secretion: Special Reference to Expression of Glucose Transporter Isoforms. Endocrinology.

[160] Park S., Ahn I.S., Kwon D.Y., Ko B.S., Jun W.K. (2008). Ginsenosides Rb1 and Rg1 Suppress Triglyceride Accumulation in 3T3-L1 Adipocytes and Enhance β-Cell Insulin Secretion and Viability in Min6 CellsviaPKA-Dependent Pathways. Biosci. Biotechnol. Biochem..

[161] Yaluri N., Modi S., Rodríguez M.L., Stancáková A., Kuusisto J., Kokkola T., Laakso M. (2015). Simvastatin Impairs Insulin Secretion by Multiple Mechanisms in MIN6 Cells. PLoS ONE.

[162] Bhat U.G., Ilievski V., Unterman T.G., Watanabe K. (2014). Porphyromonas gingivalis Lipopolysaccharide Upregulates Insulin Secretion From Pancreatic β Cell Line MIN6. J. Periodontol..

[163] Vasu S., McClenaghan N.H., McCluskey J.T., Flatt P.R. (2013). Cellular responses of novel human pancreatic β-cell line, 1.1B4 to hyperglycemia. Islets.

[164] Green A.D., Vasu S., McClenaghan N.H., Flatt P.R. (2016). Implanting 1.1B4 human β-cell pseudoislets improves glycaemic control in diabetic severe combined immune deficient mice. World J. Diabetes.

[165] Vasu S., McClenaghan N.H., Flatt P.R. (2016). Molecular Mechanisms of Toxicity and Cell Damage by Chemicals in a Human Pancreatic Beta Cell Line, 1.1B4. Pancreas.

[166] Vasu S., McClenaghan N.H., McCluskey J.T., Flatt P.R. (2013). Effects of lipotoxicity on a novel insulin-secreting human pancreatic β-cell line, 1.1B4. Biol. Chem..

[167] Green A.D., Vasu S., McClenaghan N.H., Flatt P.R. (2015). Pseudoislet formation enhances gene expression, insulin secretion and cytoprotective mechanisms of clonal human insulin-secreting 1.1B4 cells. Eur. J. Physiol..

[168] Ravassard P., Hazhouz Y., Pechberty S., Bricout-Neveu E., Armanet M., Czernichow P., Scharfmann R. (2011). A genetically engineered human pancreatic β cell line exhibiting glucose-inducible insulin secretion. J. Clin. Investig..

[169] Tsonkova V.G., Sand F.W., Wolf X.A., Grunnet L.G., Ringgaard A.K., Ingvorsen C., Winkel L., Kalisz M., Dalgaard K., Bruun C. (2018). The EndoC-βH1 cell line is a valid model of human beta cells and applicable for screenings to identify novel drug target candidates. Mol. Metab..

[170] Brozzi F., Nardelli T.R., Lopes M., Millard I., Barthson J., Igoillo-Esteve M., Grieco F.A., Villate O., Oliveira J.M., Casimir M. (2015). Cytokines induce endoplasmic reticulum stress in human, rat and mouse beta cells via different mechanisms. Diabetologia.

[171] Teraoku H., Lenzen S. (2017). Dynamics of Insulin Secretion from EndoC-βH1 β-Cell Pseudoislets in Response to Glucose and Other Nutrient and Nonnutrient Secretagogues. J. Diabetes Res..

[172] Scharfmann R., Didiesheim M., Richards P., Chandra V., Oshima M., Albagli O. (2016). Mass production of functional human pancreatic β-cells: Why and how?. Diabetes Obes. Metab..

